# Optimizing Interdisciplinary Referral Pathways for Chronic Obstructive Pulmonary Disease Management Across Cardiology and Pulmonology Specialties in the Kingdom of Saudi Arabia

**DOI:** 10.3390/jcm14248865

**Published:** 2025-12-15

**Authors:** Majdy M. Idrees, Yahya Z. Habis, Ibrahim Jelaidan, Waleed Alsowayan, Osama Almogbel, Abdalla M. Alasiri, Faisal Al-Ghamdi, Abeer Bakhsh, Faris Alhejaili

**Affiliations:** 1 Division of Pulmonary Medicine, Department of Medicine, Prince Sultan Military Medical City, P.O. Box 7897, Riyadh 11159, Saudi Arabia; 2 Pulmonology Division, Department of Internal Medicine, Faculty of Medicine, Saudi Arabia & King Abdulaziz University Hospital, King Abdulaziz University, P.O. Box 80200, Jeddah 21589, Saudi Arabia; 3 King Abdullah International Medical Research Center, College of Medicine—Western Region, King Saud Bin Abdulaziz University for Health Sciences, Ministry of National Guard Health Affairs, P.O. Box 9515, Jeddah 21423, Saudi Arabia; jelaidanim@mngha.med.sa; 4 Security Forces Hospital Program, Division of Pulmonary Medicine, Department of Medicine, P.O. Box 3643, Riyadh 11481, Saudi Arabia; waas55@hotmail.com; 5 Cardiac Sciences Department, King Fahad Cardiac Center, King Saud University, P.O. Box 7805, Riyadh 11472, Saudi Arabia; 6 Respiratory and Tuberculosis Unit, Pulmonary Medicine, Ministry of Health, Abha 62522, Aseer Province, Saudi Arabia; abokhogmah@hotmail.com; 7 King Fahad Medical City, P.O. Box 59046, Riyadh 11525, Saudi Arabia; 8 King Abdullah Medical City, P.O. Box 57657, Makkah 21955, Saudi Arabia; 9 Pulmonology Division, Department of Medicine, Saudi Arabia & King Abdulaziz University Hospital, King Abdulaziz University, P.O. Box 80200, Jeddah 21589, Saudi Arabia

**Keywords:** COPD, interdisciplinary care, referral pathways, cardiology, pulmonology

## Abstract

**Background:** Chronic obstructive pulmonary disease (COPD) is a progressive respiratory condition with significant economic burden, morbidity, and mortality rates worldwide. In the Kingdom of Saudi Arabia (KSA), 4.2% of adults 40 years and older have COPD, with a higher prevalence in men and older populations. Key risk factors include smoking, air pollution, occupational exposures, and genetics. COPD coexists with cardiovascular disease (CVD) often, making diagnosis and management more difficult. This study proposes two referral algorithms to optimize care for COPD patients with coexisting CVD in the KSA. **Methods:** A nine-member cardiopulmonary task force reviewed pertinent literature, guidelines, and held virtual meetings from April to August 2025. Every algorithmic component was iteratively refined; consensus was reached when at least 80% of participants agreed, and items not reaching this threshold were revised until full agreement was reached. **Results:** According to the cardiology-to-pulmonology algorithm, patients who have unidentified respiratory symptoms or COPD risk factors undergo spirometry assessment and, if confirmed, are referred to pulmonology for diagnostic confirmation, phenotyping, and treatment, including triple fixed-dose combination therapy (TFDC) when necessary. On the other hand, the pulmonology-to-cardiology algorithm directs the evaluation of CVD risk factors and comorbidities using clinical evaluation, electrocardiogram, echocardiography, and biomarker testing, for cardiology referral. **Conclusions:** By establishing bidirectional referral pathways, morbidity and healthcare burden can be decreased, early detection can be improved, and multidisciplinary management can be strengthened. Future research should assess the feasibility, cost-effectiveness, and real-world impact within KSA’s healthcare system.

## 1. Introduction

### 1.1. Global Burden of COPD

Chronic obstructive pulmonary disease (COPD), according to the Global Initiative for Chronic Obstructive Lung Disease (GOLD) 2025, is a progressive, complex condition characterized by persistent respiratory symptoms such as breathlessness, chronic cough, sputum production, and recurrent exacerbations [1]. These clinical features result in largely irreversible airflow limitation arising from structural abnormalities of the airways, such as bronchitis and bronchiolitis, or from alveolar destruction in emphysema [1]. Spirometry is used to diagnose COPD, and a post-bronchodilator FEV_1_/FVC ratio of less than 0.7 indicates the persistent airflow obstruction [1]. Additionally, GOLD 2025 identifies two at-risk groups. Pre-COPD, characterized by symptoms or lung changes without obstruction, and preserved ratio impaired spirometry (PRISm), which is defined by decreased FEV_1_ despite a preserved ratio [1].

COPD is a significant global health concern. In 2021, it caused an estimated 3.7 million deaths and affected over 213 million people [2]. It ranks among the leading causes of disability [2]. The highest disability-adjusted life year (DALY) burdens were reported in South and East Asia, while the Middle East and North Africa (MENA) region contributes to over 2.5 million DALYs annually [2]. According to the BREATHE study, COPD prevalence in the MENA region is 3.6%, with higher rates among men [3,4]. Hospitalizations are the main contributors to healthcare use and costs in this region [4]. Ruan et al. (2023) reported that 31% of patients were readmitted within 90 days of discharge and 18% within 30 days following a COPD exacerbation [5]. Between 2020 and 2050, the global economic burden of COPD is projected to exceed INT$4 trillion, with the MENA region contributing roughly INT$103 billion [6]. Due to this growing burden, the World Health Organization (WHO), 2023, has prioritized COPD within its package of essential noncommunicable disease interventions (PEN) to promote prevention, early diagnosis, and cost-effective care, especially in low-resource settings [7].

### 1.2. Epidemiology and Economic Burden in the Kingdom of Saudi Arabia (KSA)

COPD presents a growing public health challenge in KSA. Al-Jahdali et al. (2025) [4] reported a COPD prevalence of 4.2% among adults aged 40 years and older, higher in men (5.7%) than in women (2.5%). The prevalence increased with age (11.4% among those aged 60 years and older) and smoking exposure (10.5% among individuals with more than 20 pack-years) in KSA [4]. Between 1990 and 2019, Feizi et al. (2022) documented a 49% increase in age-standardized COPD prevalence, rising from 1381 to 2053 per 100,000 population, which is one of the largest increases observed in the MENA region [8]. In 2019, an estimated 434,561 people were living with COPD in KSA, with rates slightly higher in men than in women (2124 vs. 1976 per 100,000) [8,9].

The economic burden is substantial. In 2019, the direct medical costs of seven major noncommunicable diseases (NCDs) across Gulf Cooperation Council (GCC) countries reached INT$16.7 billion, equivalent to 0.6% of the region’s gross domestic product (GDP). In KSA, COPD accounted for approximately 15.6% of this spending on major NCDs, representing a direct medical cost burden of INT$1320 million, exceeding that of asthma [10,11]. Healthcare spending on major NCDs in KSA is projected to increase from USD 19.8 billion in 2020 to USD 32.4 billion by 2030. COPD prevalence in KSA is projected to rise from 1.8% in 2020 to 2.5% in 2030, with a per-patient annual cost of USD 5913 in 2020 [12,13]. Productivity losses further add to this burden across GCC countries. NCD-related absenteeism and presenteeism account for 0.5% and 2.2% of GDP, respectively [10], while in KSA, combined healthcare and productivity costs total USD 18.6 billion annually (2.8% of GDP) [14].

### 1.3. Risk Factors for COPD

The GOLD 2025 report identifies several risk factors for COPD, including tobacco smoking, biomass fuel and air pollution exposure, occupational dust and chemicals, and genetic predispositions such as alpha-1-antitrypsin deficiency [1]. Additional risk factors include low birth weight, impaired lung development, accelerated lung aging, and socioeconomic disadvantage, with cumulative lifetime exposures, referred to as GETomics (Genetics × Environment × Time), as central to disease pathogenesis [1]. Adeloye, D. et al. (2022) confirmed that the COPD risk is associated with smoking, being male, low body mass index, household air pollution, and occupational exposures [15]. WHO reported that more than 70% of COPD cases in high-income nations are caused by tobacco smoking [16]. On the other hand, tobacco smoking causes 30–40% of COPD cases in low- and middle-income countries, with indoor air pollution being a significant risk factor [16].

In the KSA, the Saudi Thoracic Society (STS) COPD guideline reported cigarette and waterpipe (shisha) smoking as a significant risk factor, along with biomass fuel, air pollution, occupational exposures, and prior respiratory illness such as asthma or tuberculosis [4]. Studies in KSA demonstrated impaired lung function and systemic inflammation in long-term cigarette and shisha smokers [17], while vaping has also been associated with persistent respiratory symptoms [18]. These risks are further exacerbated by sedentary lifestyles and limited awareness about chronic respiratory conditions [19], with national survey data showing that more than three-quarters of adults and most smokers had never heard of COPD [20].

### 1.4. COPD and Cardiovascular Comorbidities

COPD frequently coexists with cardiovascular diseases (CVDs), such as heart failure (HF), ischemic heart disease, pulmonary hypertension (PH), and arrhythmias, due to shared risk factors such as smoking, aging, and systemic inflammation [4]. HF occurs in over 20% of COPD patients, a 4.5-fold higher risk than in non-COPD populations, with COPD present in 20–32% of HF cases [4]. PH is caused by chronic hypoxia-induced vasoconstriction and vascular remodeling, with prevalence ranging from 6–60%. Although severe PH is uncommon, it occurs in 1–3.7% of cases. [4]. Arrhythmias, especially atrial fibrillation, are common and associated with reduced FEV_1_, yet generally do not necessitate modification of standard COPD therapy [4].

Rogliani et al. (2021) and Miklos & Horvath (2023) emphasized the bidirectional link between COPD and CVD, driven by inflammation, oxidative stress, and endothelial dysfunction, which worsen both cardiac and respiratory outcomes [21,22]. Within this overlap, about 70% of patients have HF with preserved ejection fraction (HFpEF) and 20% have HF with reduced ejection fraction (HFrEF) [23]. HFrEF is associated with more HF-specific hospitalizations and higher mortality, while HFpEF correlates more with COPD exacerbations [23]. Preiss et al. (2025) showed that hypoxia reduces right ventricle (RV) strain in COPD from −26% to −23.9%, revealing subclinical RV dysfunction [24]. Thus, RV impairment, secondary to left ventricle (LV) failure or primary to hypoxic COPD, signals poor prognosis and underscores the need for echocardiography with strain analysis and coordinated cardio-pulmonary management.

### 1.5. COPD Exacerbations and CVD Outcomes

**Figure 1:**
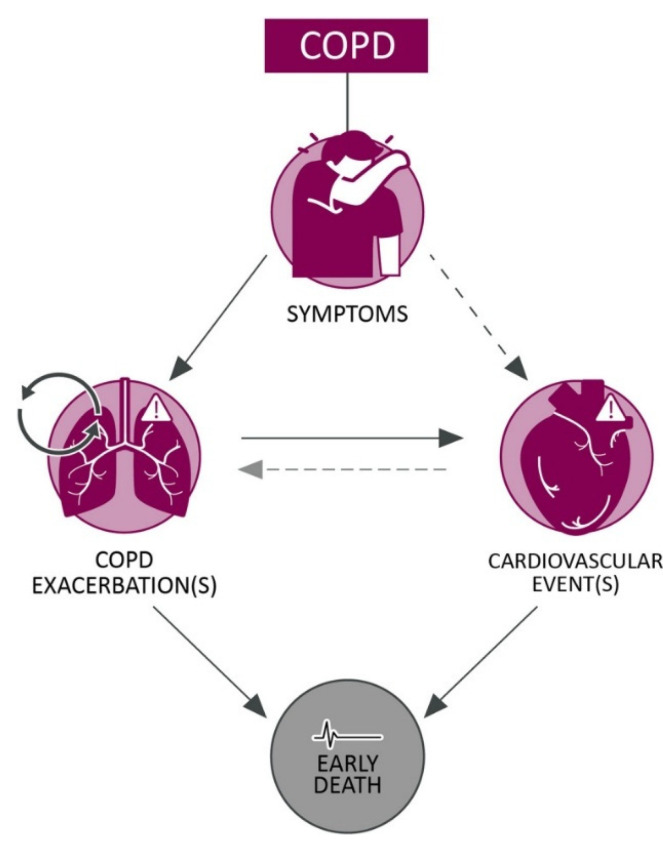
COPD-associated cardiopulmonary risk. Arrow type and shade indicate strength of association: strong association, with substantial supporting data (dark grey solid), emerging association, with some supporting data (dark grey dotted), suspected association, with data yet to be generated (light grey dotted). COPD: chronic obstructive pulmonary disease. (Reproduced from [25]. Reprinted with permission from Springer Nature. Licensed under CC BY-NC 4.0 https://creativecommons.org/licenses/by-nc/4.0/ (accessed on 10 August 2025)).

COPD exacerbations significantly increase the risk of cardiovascular complications and long-term mortality. Singh et al. (2024) [25] illustrate the bidirectional relationship between COPD exacerbations and cardiopulmonary events. Progressive COPD is characterized by frequent and severe exacerbations, which cause systemic inflammation and physiological stress, thereby accelerating the decline in lung function and elevating the risk of cardiovascular complications, such as myocardial infarction, stroke, and heart failure (Figure 1) [25].

Large-scale studies highlighted the cardiovascular risk in COPD. Sa Sousa et al. (2024) analyzed a cohort of over 3.4 million COPD patients and reported a higher incidence of CVD [26]. Similarly, Hadi et al. (2010) reported that in the Middle East, 5.2% of patients hospitalized with acute coronary syndrome (ACS) also had COPD, and these patients experienced increased rates of heart failure, stroke, and in-hospital complications [27]. EXACOS-CV program (Vogelmeier et al., 2024; Santos et al., 2025) demonstrated that cardiovascular risk increases sixteenfold in the first week after a COPD exacerbation and remains elevated for more than a year [28,29].

Recurrent exacerbations and early lung function abnormalities, such as PRISm, further increase these risks. About one in four people with COPD experience recurrent exacerbations, which can hasten the deterioration of lung function [4]. On average, patients have between 0.5 and 3.5 exacerbations annually, and those who have two or more episodes a year are more likely to be hospitalized, have higher rates of morbidity, and die sooner than those who have fewer exacerbations [4]. The mortality rate for patients admitted for acute exacerbations of COPD (AECOPD) is approximately 25% at one year and 65% at five years [4]. Studies have identified PRISm as a high-risk phenotype [4]. Wijnant et al. (2020) reported that PRISm affects approximately 7.1% of adults aged 45 years or older and is associated with increased all-cause mortality (HR 1.6), higher cardiovascular mortality (HR 2.8), and accelerated FEV_1_ decline compared with normal spirometry [30]. Similarly, the NOVELTY study by Rapsomaniki et al. (2023) demonstrated that PRISm patients have significantly greater all-cause mortality than pre-COPD individuals (age-adjusted HR: 2.5; 95% CI: 1.3–4.9) [31].

**Figure 2:**
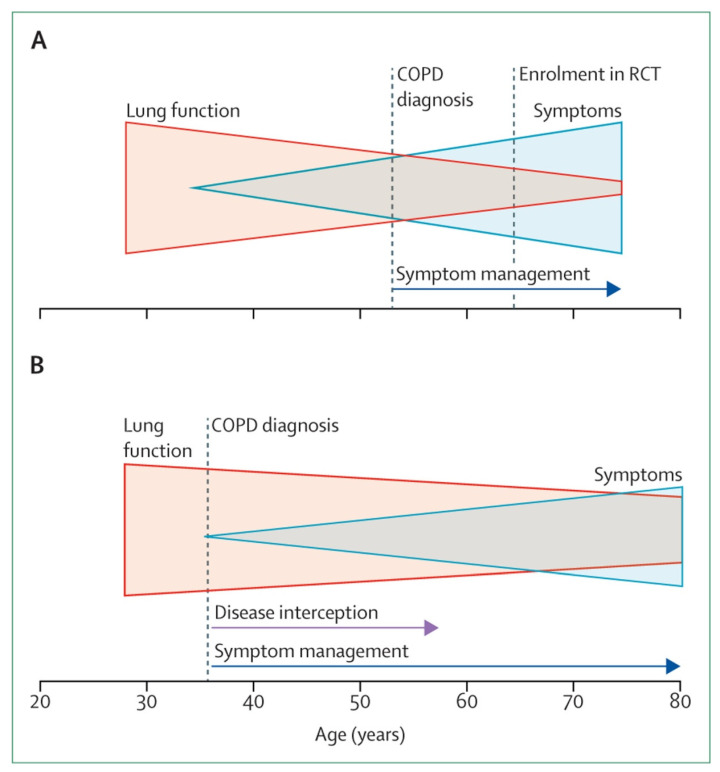
The importance of early diagnosis to eliminate COPD. (**A**) Currently, COPD is diagnosed at a stage when pathological changes are irreversible. (**B**) Implementation of a more inclusive diagnosis of COPD allows for the detection of early disease before irreversible pathological changes have occurred and could lead to disease interception. (Reproduced from [32], with permission from Elsevier).

As noted by Stolz et al. (2022) [32], COPD is frequently detected at advanced stages, when structural lung damage is irreversible, and treatment efficacy is reduced. Early symptoms are commonly misattributed to aging or coexisting conditions, while the limited sensitivity of spirometry and absence of predictive biomarkers further delay timely diagnosis [32] (Figure 2A). Stolz and colleagues also emphasized that a future-oriented diagnostic model, which integrates proactive screening of high-risk individuals with spirometry and symptom-based questionnaires, would lead to earlier detection and timely intervention [32] (Figure 2B).

Armentaro et al. (2022) [33] reported data from 749 enrolled patients, of whom 408 had a tricuspid annular plane systolic excursion (TAPSE) of 20 mm or higher, while 341 had a TAPSE of 20 mm or lower. The incidence of major adverse cardiac events (MACEs) was 1.9 per 100 patient-years in patients with TAPSE of 20 mm or higher, compared with 4.2 per 100 patient-years in those with TAPSE of 20 mm or lower. These results demonstrate that in patients with mild COPD, right ventricular dysfunction is independently associated with an increased risk of MACEs during follow-up [33]. Subsequently, in the DELIVER trial, 11% of patients with HFmrEF or HFpEF had mild-to-moderate COPD, which was associated with a 28% higher risk of cardiovascular death or worsening heart failure compared with patients without COPD (adjusted HR: 1.28, 95% CI: 1.08–1.51) [34]. These findings highlight the importance of integrated cardiovascular and respiratory care.

### 1.6. Management of COPD and CVD Comorbidities

**Figure 3:**
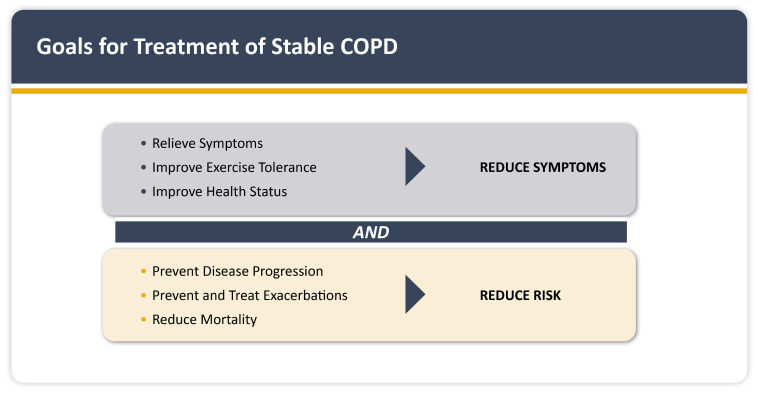
GOLD 2025 treatment goals in stable COPD (Figure reproduced with permission: 2024, 2025 Global Initiative for Chronic Obstructive Lung Disease, available from www.goldcopd.org, published in Deer Park, IL, USA).

The GOLD guidelines support an integrated management strategy that concurrently addresses the comorbidities of CVD and COPD [1]. A thorough, integrated strategy that emphasizes symptom and risk assessment (Figure 3) as well as comorbidity management is necessary for the effective management of stable COPD [1]. Smoking cessation services are still not widely used in the KSA, which emphasizes the need for focused approaches to increase access and aid in national tobacco control initiatives [35].

**Figure 4:**
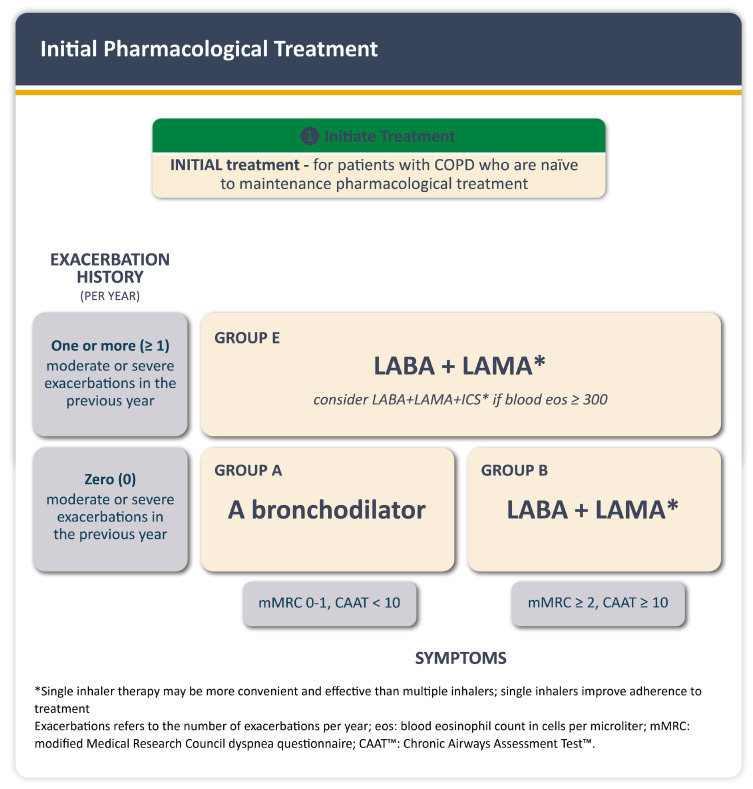
Initial pharmacological treatment in COPD (Figure reproduced with permission: 2025, 2026 Global Initiative for Chronic Obstructive Lung Disease, available from www.goldcopd.org, published in Deer Park, IL, USA).

For initial pharmacological treatment, GOLD 2026 uses the ABE classification, which is determined by symptom burden assessed with the modified Medical Research Council (mMRC) dyspnea scale, the COPD Assessment Test (CAT), and the patient’s history of exacerbations. According to (Figure 4), Group A patients are recommended a single bronchodilator, either a short or long-acting beta-agonist (LABA) or a long-acting muscarinic antagonist (LAMA). If available and affordable, a long-acting bronchodilator is the preferred choice except in patients with occasional breathlessness. Group B patients should begin with dual bronchodilation (LABA + LAMA). Group E patients, those with one or more moderate or severe exacerbations in the previous year, should receive LABA + LAMA as initial therapy, with consideration of the triple fixed dose combination (TFDC) of LABA + LAMA + inhaled corticosteroids (ICS), if blood eosinophils are greater than or equal to 300 cells/µL [36].

In the 2026 update, the GOLD A, B, and E categories were revised, as evidence from observational studies indicates that even a single moderate or severe exacerbation before initiating maintenance pharmacological therapy increases the risk of subsequent events. The threshold of one moderate exacerbation should now be used to consider treatment escalation, with blood eosinophils 100 to <300 cells/µL to achieve a low disease activity state characterized by no exacerbations [36].

**Figure 5:**
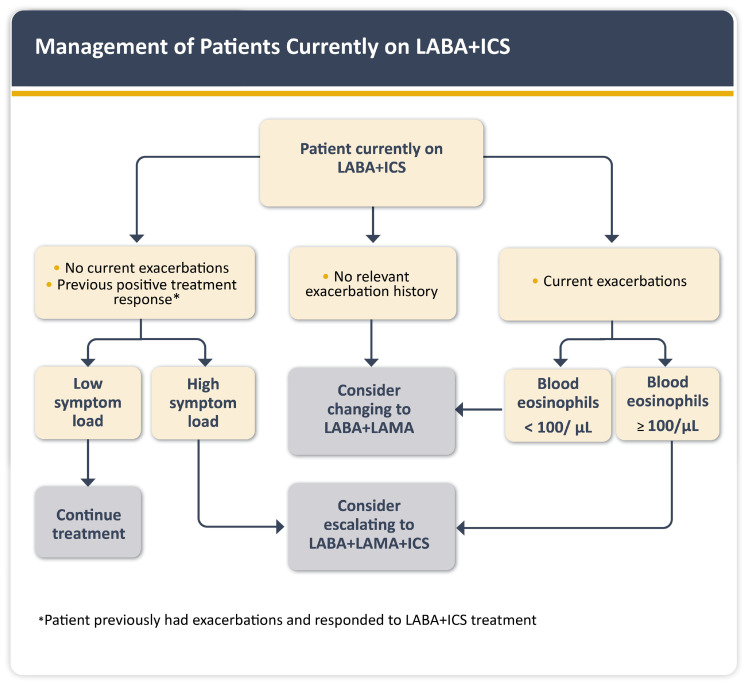
Management of patients currently on LABA + ICS (Figure reproduced with permission: 2024, 2025 Global Initiative for Chronic Obstructive Lung Disease, available from www.goldcopd.org, published in Deer Park, IL, USA).

For patients on LABA + ICS, GOLD 2025 recommends reassessing the need for ICS based on exacerbation history and symptom control. Those without prior exacerbations may be switched to LABA + LAMA, while patients with ongoing symptoms or exacerbations may benefit from TFDC. An eosinophil count of ≥100 cells/µL favors initiating ICS therapy, while counts ≥300 cells/µL strongly favor its use due to the higher likelihood of clinical benefit (Figure 5) [1,4].

One of the most important aspects of integrated COPD care is comorbidity management. In heart failure, beta-blockers are safe, but in pulmonary hypertension, oxygen and optimal lung therapy are needed. When arrhythmias occur, high-dose beta-2 agonists should be used with caution. Clinical assessment, spirometry, and echocardiography are necessary for the accurate differentiation of heart failure from COPD, with B-type natriuretic peptide (BNP) helping to differentiate cardiac events from exacerbations [4]. While the WHO functional classification (2022 ESC/ERS Guideline) standardizes grading when pulmonary hypertension is present, the New York Heart Association (NYHA) functional classification (2022 AHA/ACC/HFSA Guideline) is preferred for evaluating HF-related symptoms and exercise limitation in patients with both COPD and heart failure [37,38].

**Figure 6:**
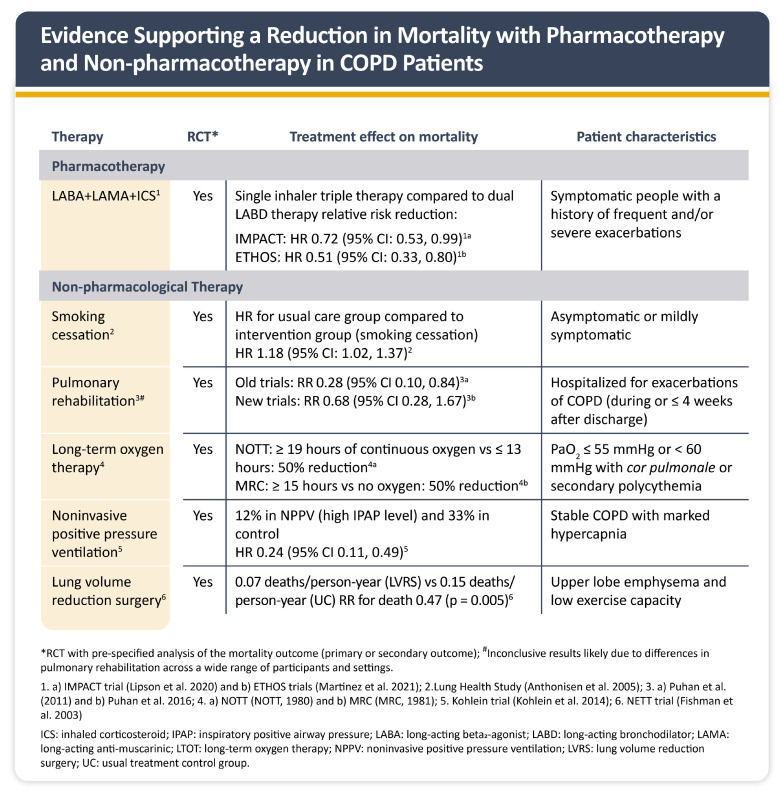
Evidence for mortality reduction with pharmacotherapy and non-pharmacotherapy in COPD patients [39,40,41,42,43,44,45,46,47]. (Figure reproduced with permission: 2024, 2025 Global Initiative for Chronic Obstructive Lung Disease, available from www.goldcopd.org, published in Deer Park, IL, USA).

As shown in Figure 6, TFDC therapy has demonstrated significant benefits, reducing all-cause mortality (IMPACT HR 0.72; ETHOS HR 0.51) [1]. The Saudi COPD task force confirmed that TFDC therapy reduces both moderate-to-severe (RR 1.16) and severe exacerbations (RR 1.22) [4]. These findings support the use of TFDC therapy in high-risk patients to improve exacerbation control, reduce mortality, and enhance overall disease management. To further improve survival, quality of life, and overall disease outcomes, non-pharmacologic interventions such as pulmonary rehabilitation, long-term oxygen therapy, and smoking cessation remain essential (Figure 6) [1].

The six-minute walk distance (6MWD) is a simple and reliable measure of exercise capacity. In KSA, Shaphe et al. (2025) reported that 35 patients with COPD walked an average of 382 m ± 65 m, showing that the test is safe and feasible [48]. Alshammri et al. (2024) recommended using the 6MWD to guide individualized exercise plans in COPD rehabilitation programs in KSA [49]. Alahmadi (2025) reported that pulmonary rehabilitation can improve 6MWD by 113–300 m. As most evidence comes from a single center in KSA, it is promising, yet highlights the urgent need to expand such services nationwide [50].

Although treatment plans are well-defined, prompt diagnosis and well-coordinated multidisciplinary care are essential for their successful implementation, and these aspects of clinical practice in the KSA are still lacking.

### 1.7. Diagnostic Challenges, Care Gaps, and Rationale for Bi-Directional Referral Pathways

Diagnosing COPD remains challenging due to overlapping symptoms, limited use of diagnostic tools, and fragmented care pathways [20]. In the KSA, 56% of people with previous respiratory diagnoses have never had spirometry, and only 16% of symptomatic individuals receive consultation or pulmonary function testing [20]. Thomas et al. (2019) and Canepa et al. (2019) showed that relying on a fixed FEV_1_/FVC ratio (<0.7) can lead to both an under- and an overdiagnosis, while comorbidities like asthma and heart failure frequently mimic COPD, making assessment even more difficult [51,52]. Tash and Al-Bawardy (2023) highlighted that systemic limitations, fragmented healthcare, poor coordination between referral centers, and limited primary care capacity undermine care quality and preventive efforts within KSA [53]. In KSA, Aljerian et al. (2024) evaluated more than 600,000 interspecialty referrals captured within the national Saudi medical appointments and referrals centre (SMARC) system, demonstrating that cardiology–pulmonology referrals accounted for 62.5% of all cases [54].

Despite these overlaps, referral practices between pulmonology and cardiology in the KSA remain inconsistent, reactive, and largely unstandardized. Currently, there is no clear framework or criteria to guide timely referrals for patients with coexisting respiratory and cardiovascular symptoms. As a result, referrals often occur only after acute events, without structured feedback or coordination, leading to delayed diagnoses, fragmented care, and redundant investigations. This consensus initiative aims to address these gaps by developing structured, evidence-based, context-specific bidirectional referral algorithms to promote proactive, coordinated management and improve outcomes for high-risk patients.

The GOLD 2025 report refines spirometry interpretation for COPD diagnosis, emphasizing the complementary roles of pre- and post-bronchodilator testing. The report also recommends using updated reference standards, such as the lower limit of normal (LLN) or z-scores, as part of improved interpretative guidance to minimize age-related misclassification [1]. Additionally, GOLD 2025 highlights that CVDs are among the most common and clinically significant comorbidities in patients with COPD, stressing that these conditions should be identified and treated according to established cardiovascular guidelines, regardless of the presence of COPD, thereby promoting proactive investigation and therapeutic intervention [1]. Regional evidence supports this: Shehab et al. (2025) developed and implemented a UAE-specific cardiology-to-pulmonology referral algorithm designed to enhance early identification of COPD in CVD patients and to promote coordinated management [55]. Similarly, recent global consensus statements by Shrikrishna et al. (2025) [56] and Gale et al. (2025) [57] emphasize the importance of systematically assessing cardiopulmonary risk in patients with COPD. They recommend appropriate screening, including spirometry in at-risk individuals, and stress that cardiopulmonary complications are frequently underrecognized and undertreated. Both statements advocate closer collaboration between respiratory, cardiac, and primary care teams to improve outcomes and reduce morbidity and mortality in this population [56,57].

Therefore, we convened a multidisciplinary task force to develop bidirectional referral algorithms specific to the KSA healthcare system, aiming to bridge the critical gap in current practice and facilitate proactive, integrated cardiopulmonary care.

## 2. Materials and Methods

The task force comprised nine experts: five pulmonologists and four cardiologists. The selection of taskforce members was based on predefined criteria, which included recognized clinical expertise in COPD and CVD, leadership roles in national healthcare institutions or professional societies, and demonstrated academic contributions such as peer-reviewed publications or participation in guideline development. Expert selection was coordinated by the task force coordinator to ensure balanced representation between cardiology and pulmonology, as well as across different healthcare settings.

Between April and August 2025, the task force group held virtual consensus meetings. The initial meeting focused on the disease burden and care gaps, review of current evidence and GOLD 2025 updates, identification of diagnostic and referral barriers, and development of referral algorithms. Evidence sources included national and international epidemiological data, current COPD and CVD guidelines, and supporting peer-reviewed studies. Draft referral algorithms were developed after the first meeting and refined through successive rounds of discussion. Consensus discussions followed a stepwise approach in which proposed items were reviewed during meetings and, if needed, refined between meetings. Iterative feedback and revisions were exchanged by email to optimize clinical clarity and real-world applicability. Final agreement on both referral algorithms was achieved by consensus during the fourth meeting. Each item of the referral algorithms was iteratively reviewed and refined, with at least 80% of the task force members required to agree for initial consensus. Items that did not reach this threshold were revised until all members agreed.

The final outputs comprised two referral algorithms:

- Cardiology-to-Pulmonology referral algorithm to guide cardiologists/non-pulmonologists in identifying patients with suspected or undiagnosed COPD.
- Pulmonology-to-Cardiology referral algorithm to guide pulmonologists in evaluating cardiovascular risk and referring patients for cardiac evaluation.

## 3. Results

### 3.1. Cardiology-to-Pulmonology Referral Algorithm

Clinical scenario 1: A 60-year-old male smoker with diabetes presents to the cardiology clinic with acute chest pain. He is diagnosed with acute coronary syndrome. After stabilization, he reports chronic cough and intermittent wheezing. Spirometry reveals airflow limitation consistent with COPD. He is referred to pulmonology for further assessment and initiation of treatment.

**Figure 7:**
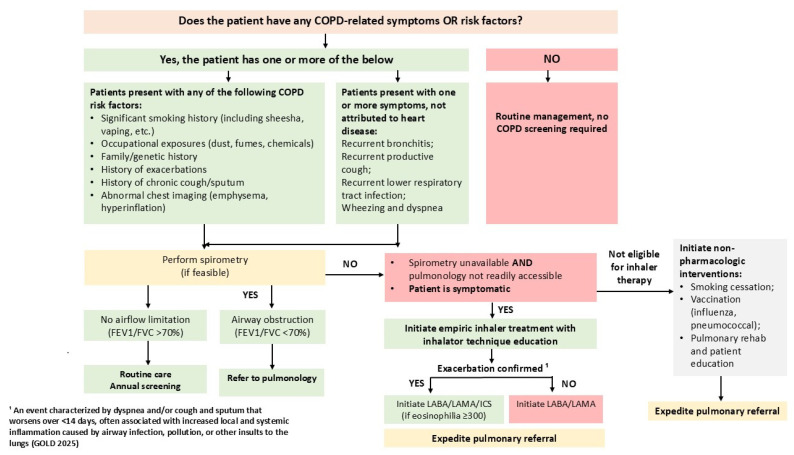
Cardiology/non-pulmonology to pulmonology clinic referral algorithm. This figure outlines a simple approach for cardiologists and non-pulmonologists to identify possible COPD and determine when to arrange testing, start initial treatment, or refer to pulmonology.

This scenario illustrates the cardiology-to-pulmonology referral algorithm (Figure 7), which facilitates early recognition of COPD in cardiology settings in the KSA.

The algorithm begins with identifying patients with COPD risk factors such as a smoking history (cigarettes, shisha, vaping), occupational or environmental exposure, recurrent exacerbations, chronic cough or sputum, abnormal chest imaging, or a family history of respiratory disease. When symptoms are not attributed to CVD, such as recurrent bronchitis, productive cough, wheezing, dyspnea, or recurrent lower respiratory tract infections, spirometry is recommended to assess for airflow obstruction (post-bronchodilator FEV_1_/FVC < 0.70).

If airway obstruction is present, patients are referred to pulmonology for confirmation, phenotyping, and initiation of treatment.

If spirometry results are normal, routine annual screening is recommended for individuals at high risk.

In symptomatic patients where spirometry and pulmonology services are not immediately available, empiric inhaler therapy with dual long-acting bronchodilators (LABA/LAMA) may be initiated, along with proper inhaler technique education. If the patient is not suitable for inhaled therapy, non-pharmacologic interventions should be implemented. These include smoking cessation, referral to pulmonary rehabilitation, and self-management education. Those with one or more moderate or severe exacerbations in the previous year should start treatment with LABA + LAMA, with escalation to TFDC of LABA + LAMA + ICS when blood eosinophil counts are ≥300 cells/µL. Urgent referral to pulmonology is warranted for these patients.

### 3.2. Pulmonology-to-Cardiology Referral Algorithm

Clinical scenario 2: A 58-year-old male smoker with a history of hypertension and dyslipidemia presents with intermittent cough and dyspnea that worsen with exertion. He also reports intermittent chest discomfort. Spirometry confirms moderate COPD. Baseline cardiac testing is performed, including electrocardiogram (ECG), echocardiography, and N-terminal pro-B-type natriuretic peptide (NT-proBNP). Abnormal findings prompt referral to cardiology for evaluation and co-management.

**Figure 8:**
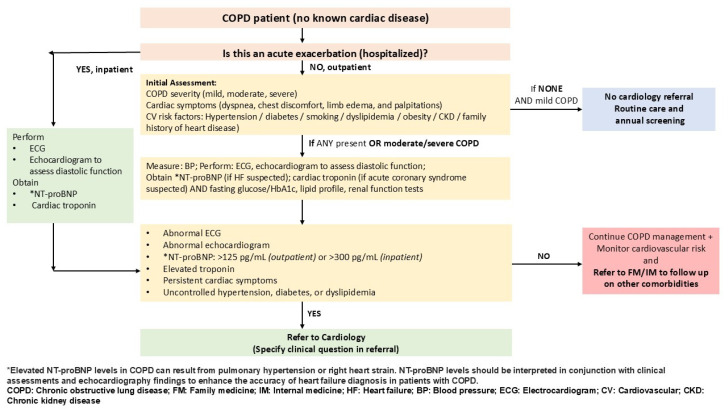
COPD-specific algorithm: pulmonology clinic to cardiology clinic referral. This algorithm guides pulmonologists on when to screen for CVD and when to refer patients with COPD for cardiology evaluation, based on symptoms, disease severity, and cardiac risk markers.

This case illustrates the pulmonology-to-cardiology referral algorithm (Figure 8), which helps pulmonologists identify COPD patients with possible cardiovascular comorbidities and refer them to a cardiologist.

After confirming COPD, clinicians should determine the severity of the disease as mild, moderate, or severe using spirometry, symptoms, and exacerbation history. The next step is to check for cardiovascular symptoms, such as exertional fatigue, orthopnea, palpitations, chest discomfort, or peripheral edema. Clinicians should also review cardiovascular risk factors, including diabetes, hypertension, dyslipidemia, smoking, obesity, chronic kidney disease, and family history of heart disease.

For mild COPD without risk factors, annual cardiovascular screening (blood pressure, lipid profile, glucose/HbA1c) is recommended.

For moderate-to-severe COPD or those with risk factors, baseline cardiac investigations are advised, including 12-lead ECG, echocardiography, fasting lipid profile, fasting glucose/HbA1c, and NT-proBNP measurement.

Cardiology referral is triggered by NT-proBNP > 125 pg/mL, abnormal ECG or echocardiographic findings, or uncontrolled comorbidities.

In the inpatient setting, particularly during acute exacerbations, NT-proBNP >300 pg/mL or elevated cardiac troponin suggests possible heart failure and warrants urgent cardiology referral.

The two referral algorithms complement each other in guiding patient management. In routine practice, diagnosing the two conditions can be challenging because symptoms such as dyspnea, fatigue, cough, chest discomfort, and persistent wheeze frequently overlap. For patients seen in cardiology clinics, persistent respiratory symptoms that cannot be attributed to cardiac status should prompt COPD screening with spirometry and consultation with a pulmonologist. In a pulmonology practice, active cardiac symptoms, elevated cardiac biomarkers, or an abnormal echocardiogram should prompt consideration of CVD and referral to a cardiologist. In both specialties, early detection enables timely referral and focused evaluation. It is possible to determine the cause of symptoms and guide the right course of treatment by collaboratively interpreting results from NT-proBNP, echocardiography, and spirometry. This coordinated approach encourages integrated care, minimizes repetitive investigations, and supports prompt diagnosis for patients with overlapping COPD and CVD.

## 4. Discussion

The bidirectional referral pathways proposed in this study support the prompt identification, risk assessment, and intervention, while facilitating the integrated, early management of patients with cardiovascular comorbidities and COPD. In accordance with GOLD 2025 guidelines, these pathways standardize screening, evaluation, and therapeutic decision-making, and encourage coordinated multidisciplinary care [1].

According to GOLD 2025 and the STS COPD guideline, the cardiology-to-pulmonology pathway ensures that patients with COPD risk factors or unexplained respiratory symptoms receive early spirometry, prompt referral, and initiation of TFDC therapy, when necessary [1,4]. Routine follow-up and annual screening are emphasized to enable early detection and tailored management. This aligns with the findings of Di Chiara et al. (2025), which indicate that early intervention, optimized therapy, patient education, and multidisciplinary care effectively reduce exacerbations and hospital readmissions, ultimately improving patient outcomes [58]. Additionally, Zheng et al. (2018) and Al-Jahdali et al. (2025) indicate that TFDC therapy is more effective than dual or monotherapy in reducing exacerbations [4,59]. Zheng et al. (2028) observed that TFDC therapy provides minimal survival advantages but is linked to an increased risk of pneumonia. This indicates that TFDC is best suited for patients with more severe COPD symptoms that are not sufficiently managed by dual therapy [59]. The algorithms’ recommendations are consistent with the findings of Stolz et al. (2022), who advocated for early detection and a future-oriented model using spirometry and symptom-based screening to enable timely and prompt intervention, thereby mitigating disease progression [32].

The pulmonology-to-cardiology pathway systematically evaluates COPD patients for cardiovascular manifestations, risk factors, and relevant biomarkers to ensure timely referral of moderate-to-severe or high-risk cases, particularly during acute exacerbations. This approach is supported by De Miguel-Díez et al. (2024), who demonstrated that early bidirectional referral, appropriate inhaled therapy, cardioselective treatment of CVD, and biomarker utilization significantly improve outcomes [60]. Fernandes et al. (2019) further highlighted that multidisciplinary collaboration enhances diagnostic accuracy and therapeutic effectiveness in pulmonary hypertension, underscoring the importance of integrated care for COPD with cardiovascular comorbidities [61]. These recommendations are consistent with GOLD 2025, which emphasizes the under-recognition of CVD in COPD and calls for proactive cardiovascular screening, biomarker-guided assessment, and Guideline-Directed Medical Therapy (GDMT) within a multidisciplinary framework [1].

In KSA, CVD risk assessment is an essential component of COPD care, which calls for individualized treatments, coordinated management, and patient education tailored to the country’s healthcare system [4]. The National Guidelines Program of the Ministry of Health collaborated to develop the Saudi Thoracic Society COPD guidelines, intended to be nationally applicable and aligned with the objectives of the Vision 2030 healthcare transformation [4]. According to Tash and Al-Bawardy (2023), the relevance is emphasized, given the rising burden of obesity, hypertension, diabetes, dyslipidemia, and sedentary lifestyles, which significantly increase cardiovascular morbidity, mortality, and healthcare costs in the KSA [53], while fragmented healthcare delivery further undermines outcomes [53]. Vision 2030 initiatives, including public awareness campaigns, national CVD registries, and digital health infrastructure, support the prevention and early management of COPD and CVD [53]. Accordingly, the proposed bidirectional referral algorithms are explicitly designed to be integrated within the KSA healthcare system, leveraging these existing national initiatives to improve COPD–CVD outcomes. Although the framework may be adaptable to other healthcare settings, its structure and implementation priorities are optimized for KSA-specific needs.

Our referral framework builds on prior models, including the UAE-specific algorithm developed by Shehab et al. (2025), which focused primarily on referrals from cardiology to pulmonology [55]. Unlike that algorithm, our framework takes a bidirectional approach, systematically integrating pulmonology to cardiology referrals to enable early detection and management of cardiovascular comorbidities. It incorporates validated biomarkers, structured risk stratification, and continuous multidisciplinary follow-up. The model is tailored to the KSA’s healthcare infrastructure to address challenges such as limited specialist availability, fragmented care, and variable patient awareness. It specifically addresses inconsistent health literacy and delayed symptom recognition. In situations where specialist access or patient engagement is limited, this approach embeds patient-centered flexibility, allowing empiric or supportive interventions and structured follow-up. This provides a pragmatic, context-sensitive strategy aligned with the STS COPD guidelines and KSA’s vision 2030 priorities [4].

Early diagnosis remains critical, particularly in resource-constrained settings. Au-Doung et al. (2022) and Su et al. (2024) note that the Prevalence, Underdiagnosis, and Management of COPD in Latin America (PUMA) questionnaire, which integrates demographic, exposure, and symptom parameters, effectively identifies high-risk patients [62,63]. Embedding such validated tools into digital health platforms can enhance early detection, where spirometry access is limited. The WHO Global Strategy on Digital Health 2020–2025 (2021) provides a policy framework for integrating decision-support tools into national systems [64]. Solomon et al. (2023) further demonstrated the feasibility of platforms such as EvidencePoint, which can centrally host tools like the PUMA score and deploy them across diverse healthcare systems, supporting timely screening and coordinated care [65].

Despite these strengths, a key limitation of the proposed pathways is the current absence of real-world validation data. The framework has not yet been prospectively evaluated for feasibility, clinical effectiveness, or cost-efficiency within the KSA healthcare context. Potential implementation barriers include variability in primary care expertise, uneven digital infrastructure, and patient adherence. Although empiric therapy may be initiated when specialist access is limited, clinical judgment and targeted training remain essential.

Future studies should therefore focus on external validation and real-world implementation to assess patient outcomes, scalability, and cost-effectiveness, ensuring that the framework delivers measurable improvements in care quality and system efficiency. Ongoing provider education and training will be crucial for timely, coordinated, and evidence-based care.

## 5. Conclusions

The importance of multidisciplinary coordination between pulmonology and cardiology is paramount, as it enables early detection of COPD and effective management of associated cardiovascular comorbidities. Early initiation of targeted TFDC therapy mitigates severe exacerbations and reduces COPD-related mortality. At the same time, coordinated treatment planning, particularly when cardiologists have access to spirometry and can implement GDMT, ensures simultaneous optimization of respiratory and cardiac outcomes. Structured, bidirectional referral pathways further guarantee timely evaluation and intervention, promoting continuity of care and improved patient outcomes. With this, healthcare systems can advance strategic objectives, such as KSA’s Vision 2030, which aims to reduce the burden of non-communicable diseases and expand equitable access to high-quality primary healthcare. Future studies should assess the cost-effectiveness, feasibility, and real-world impact of this multidisciplinary care strategy for COPD-CVD.

## Data Availability

The original contributions presented in the study are included in the article; further inquiries can be directed to the corresponding author.

## Abbreviations

The following abbreviations are used in this manuscript:

COPD	Chronic Obstructive Pulmonary Disease
GOLD	Global Initiative for Chronic Obstructive Lung Disease
STS	Society of Thoracic Surgeons
CVD	Cardiovascular Disease
PH	Pulmonary Hypertension
HF	Heart Failure
GDP	Gross Domestic Product
DALY	Disability-Adjusted Life Year
MENA	Middle East and North Africa
PRISm	Preserved Ratio Impaired Spirometry
KSA	Kingdom of Saudi Arabia
WHO	World Health Organization
NYHA	New York Heart Association
NCD	Noncommunicable Disease
GCC	Gulf Cooperation Council
PEN	Package of Essential Noncommunicable Disease Interventions
ACS	Acute Coronary Syndrome
AECOPD	Acute Exacerbation of Chronic Obstructive Pulmonary Disease
LAMA	Long-Acting Muscarinic Antagonist
LABA	Long-Acting Beta-2 Agonist
ICS	Inhaled Corticosteroid
TFDC	Triple Fixed-Dose Combination (LAMA+LABA+ICS)
NT-proBNP	N-terminal pro-B-type Natriuretic Peptide
ECG	Electrocardiogram
UAE	United Arab Emirates
SMARC	Saudi Medical Appointments and Referrals Centre
GDMT	Guideline-Directed Medical Therapy
PUMA	Prevalence, Underdiagnosis, and Management of COPD in Latin America (questionnaire)
TAPSE	Tricuspid Annular Plane Systolic Excursion
HFmrEF	Heart Failure with Mildly Reduced Ejection Fraction
HFpEF	Heart Failure with Preserved Ejection Fraction
6MWD	6-Minute Walk Distance
MACEs	Major Adverse Cardiovascular Events
RV	Right Ventricle
LV	Left Ventricle
BP	Blood Pressure
